# Unraveling the Role of Endothelial Dysfunction in Osteonecrosis of the Femoral Head: A Pathway to New Therapies

**DOI:** 10.3390/biomedicines12030664

**Published:** 2024-03-15

**Authors:** Wenkai Shao, Ping Wang, Xiao Lv, Bo Wang, Song Gong, Yong Feng

**Affiliations:** 1 Department of Orthopedics, Union Hospital, Tongji Medical College, Huazhong University of Science and Technology, Wuhan 430000, China; 2 Department of Rehabilitation, Wuhan No. 1 Hospital, Tongji Medical College, Huazhong University of Science and Technology, Wuhan 430000, China

**Keywords:** osteonecrosis of the femoral head, endothelial dysfunction, coagulopathy, inflammation, angiogenesis

## Abstract

Osteonecrosis of the femoral head (ONFH) is a disabling disease characterized by the disruption of the blood supply to the femoral head, leading to the apoptosis and necrosis of bone cells and subsequent joint collapse. Total hip arthroplasty is not optimal since most patients are young. Multiple risk factors contribute to osteonecrosis, including glucocorticoid (GC) usage, excessive alcohol intake, hypercholesterolemia, and smoking. Continuous stimulation by many variables causes a chronic inflammatory milieu, with clinical repercussions including endothelial dysfunction, leading to thrombosis, coagulopathy, and poor angiogenesis. Immune cells are the primary regulators of inflammation. Innate and adaptive immune cells interact with endothelial cells to hinder the regeneration and repair of bone lesions. An in-depth examination of the pathological drivers of ONFH reveals that endothelial dysfunction may be a major cause of osteonecrosis. Understanding the involvement of endothelial dysfunction in the chronic inflammation of osteonecrosis could aid in the development of possible therapies. This review summarizes the role of endothelial cells in osteonecrosis and further explains the pathophysiological mechanism of endothelial dysfunction in this disease from the perspective of inflammation to provide new ideas for the treatment of osteonecrosis.

## 1. Introduction

Osteonecrosis of the femoral head is a debilitating illness caused by the gradual elimination of bone cells and bone marrow caused by insufficient blood flow to the injured subchondral bone [1]. There are approximately 20,000–30,000 new cases of osteonecrosis in the United States every year and approximately 150,000 cases in China [2,3]. Numerous risk factors, such as the use of glucocorticoids and excessive alcohol consumption, as well as certain disease states, like sickle cell disease and systemic lupus erythematosus, and treatment approaches, like radiation, chemotherapy, and hip surgery, can cause osteonecrosis to progress [4,5,6,7]. Hip preservation surgery (such as core decompression, bone grafting, and osteotomy) and a combination of medications (such as anticoagulants, fibrinolysis-enhancing drugs, vasoactive agents, and lipid-lowering therapies) can be utilized to treat early ONFH [8,9,10,11]. However, more than 80% of patients ultimately require total joint arthroplasty (THA) [12]. Although THA considerably improves patients’ lifestyles, it is not regarded as the best treatment for ONFH [13]. Consequently, it is critical to comprehend the mechanism underlying femoral head necrosis and ascertain its etiology.

Although there are many factors involved in ONFH, such as stem cell differentiation imbalance, bone remodeling dysfunction, and increased intramedullary pressure, its pathogenesis remains unclear [14]. Among these pathologies, endothelial dysfunction appears to be the most convincing [15]. Endothelial dysfunction is commonly defined as any alternations affecting the endothelium’s capacity to maintain vascular homeostasis. Subsequent alternations in hemodynamics and decreased vasodilation capability contribute to insufficient blood flow and oxygen starvation, causing ischemic injury and skeletal lesions in the femoral head, which worsen the disease by triggering a proinflammatory reaction [16]. Immune cells are attracted to the impacted region by various inflammatory mediators and chemokines, where they continuously generate harmful proteases, resulting in an excess of reactive oxygen species and greater injury to the endothelium [17]. Furthermore, endothelial dysfunction drives coagulation abnormalities in injured circulatory systems, promotes hypofibrinolysis, and impedes the clearance of metabolic wastes and the delivery of essential nutrients, which thus restrict endothelial repair and revascularization [18,19].

This review focuses primarily on the mechanisms involved in endothelial dysfunction in the pathological process of femoral head necrosis. Decreased angiogenesis, coagulation abnormalities, and chronic inflammation are all significant etiological contributors. There may be significant therapeutic promise in encouraging angiogenesis or focusing on endothelial cell repair.

## 2. Endothelial Cells Orchestrate Angiogenesis–Osteogenesis Coupling

Endothelial cells primarily line the inner surfaces of bone microvessels. A variety of physiological processes, including the blood vessel–tissue barrier, hemofiltration, the maintenance of vasomotor rhythm, the regulation of nutrient transport, and the coordination of immune effects, are involved [20,21]. Their role as a highly active metabolic and endocrine organ that secretes a range of molecules to maintain balance has come to light in recent years [22]. Vascular endothelial cells are critical for skeletal development because they coordinate multiple mechanisms. The blood vessels provide the instructional vasculature niche required for bone regeneration by serving as frameworks for cells that produce bone and matrix mineralization [23]. Endothelial cells are triggered in response to various extracellular stimuli, which help to sustain osteoprogenitor cells in the bone marrow by secreting chemicals in an endocrine way. Angiocrine mediators, along with angiokines, are substances generated by the diverse vasculature network of bones. They specifically target osteoprogenitors and osteolineage cells within the actively renewing callus. (Table 1). For example, the activation of MAPK/ERK, SMAD, Wnt/β-catenin, and cAMP signaling is facilitated by the secretion of exosomes and cytokines derived from endothelial cells, which foster the formation of osteoblasts and chondrocytes [24,25,26,27,28,29]. By modulating histone lactylation, endothelial cell-derived metabolites like lactate stimulate the osteogenic differentiation of mesenchymal stem cells [30]. This endothelial cell drives the bone remodeling process and boosts the maturation of osteoprogenitors, thereby helping to orchestrate bone–vascular interactions [31].

Based on their functional characteristics and temporal arrangement, endothelial cells in mammalian bones are divided into three main types: type H, type L, and type E. Type-H endothelial cells, which highly express CD31 and endomucin (EMCN), are mostly found in the metaphyseal region, where bone metabolism is active, and they are surrounded by osteoprogenitor cells that help the bone formation process [35]. Type-L endothelial cells, which have low expression of CD31 and EMCN, are located mainly in the diaphysis and mediate the hematopoietic process [23]. However, type-E endothelial cells exist mainly transiently in the late embryonic and early postnatal development stages to promote bone formation. These cells behave similarly to type-H blood vessels, but with the stronger expression of CD31 and the weaker expression of EMCN [36]. Localizing to regions with active bone metabolism, type-H endothelial cells are encircled by osteoprogenitors expressing runx2+, collagen 1α+, and osterix, in addition to perivascular cells expressing platelet-derived growth factor receptor-β (PDGFR-β) and neuronglial antigen 2 (NG2) [31]. These cells exhibit a strong positive correlation with osteolineage cells and synergistically promote the osteogenic process.

As precursors of endothelial cells, endothelial progenitor cells (EPCs) are derived mainly from peripheral blood and bone marrow [37]. They play a pivotal role in disorders related to endothelial damage by proliferating, migrating, and differentiating into endothelial cells, in addition to secreting a range of cytokines and vascular growth factors [38]. Generally, EPCs are separated, based on their morphological traits and culture duration, into two categories: early myeloid angiogenic cells and late endothelial colony-forming cells [39]. The former mainly express cell surface markers, including CD45, CD31, and CD14, and secrete a large amount of cytokines but have a weak ability to proliferate; the latter mainly express cell surface markers, including CD31, CD105, CD146, VE-cadherin, and von Willebrand factor, and have a relatively strong ability to proliferate and differentiate into mature endothelial cells [40]. Damaged EPCs are often associated with adverse cardiovascular outcomes. Several studies have revealed that patients with GC-induced osteonecrosis of the femoral head (GIONFH) had a decrease in the number and function of circulating EPCs, as indicated by increased injury migration ability, reduced angiogenesis, and increased senescence [41,42,43]. Local hypoxia after ischemia stimulates the HIF-1α/VEGF pathway, which promotes the migration of bone marrow-derived EPCs and induces endothelial cell proliferation, migration, and differentiation [44]. Therefore, it is vital to research the causes and effects of aberrant angiogenesis or endothelial damage during ischemia, as well as the risk of postischemic osteonecrosis.

## 3. Endothelial Dysfunction Promotes Coagulopathy

The pathological characteristic of osteonecrosis caused by vascular ischemia is significantly impacted by coagulopathy [45]. Inadequate fibrinolysis and excessive thrombophilia are the primary causes of this. Both acquired and hereditary thrombophilia/hypofibrinolysis can result in osteonecrosis of the femoral head and jaw at all ages [46,47]. Extreme thrombophilia, also known as hypercoagulability, is a coagulation disorder in which a great number of blood clots form on the walls of circulating blood vessels. Fibrinolysis is the decomposition of a thrombosis or clot, and it is tightly controlled by tissue factor plasminogen inhibitor (TFPI), plasminogen activators, plasminogen activator inhibitor-1 (PAI-1), and plasmin [48]. A healthy endothelium constitutes an anticoagulant and antithrombotic protective layer and secretes an abundance of substances that directly regulate platelet activity, including nitric oxide (NO), prostaglandins, and ectonucleoside triphosphate diphosphate hydrolase-1 (E-NTPDase1) [49,50]. Endothelial cells express TFPI, which limits the effects of tissue factor (TF) and inhibits the TF-mediated overactivation of clotting factors VII and X [51]. The surfaces of endothelial cells bind to antithrombin, which effectively inhibits platelet activity. Endothelial cells generate thrombomodulin (TM) which binds to thrombin, inducing an alteration in structure that increases its affinity for protein C/S and inhibits clotting factors Va and VIIIa [52]. In addition, endothelial-derived tissue plasminogen activator (t-PA) and urokinase-type plasminogen activator (u-PA) can interact with PAI-1 to switch plasminogen into plasmin, thereby facilitating the degradation of fibrin [53].

In addition to releasing molecules that regulate coagulation and fibrinolysis, endothelial cells have autocrine and paracrine functions which produce vasoactive substances, such as NO, adenosine diphosphate (ADP), platelet-activating factor (PAF), endothelin 1 (ET-1), thrombin, and thromboxane A2 (TXA2) [54]. These substances are secreted to control vasoconstriction and platelet activation during clotting circumstances. After 24 h of GC administration, Lu et al. found that ET-1 receptor and PAI-1 expression increased dramatically, whereas NO, PGI2 synthase, PGE synthase, the PGE receptor, and ET-1 expression dropped markedly [55]. Therefore, vasoconstriction and thrombosis are promoted after GC-induced damage to endothelial cells. Reduced vasodilator release and increased vasoconstrictor production are key features of endothelial dysfunction, which is a major factor in coagulopathy and the development of ONFH.

**Figure 1:**
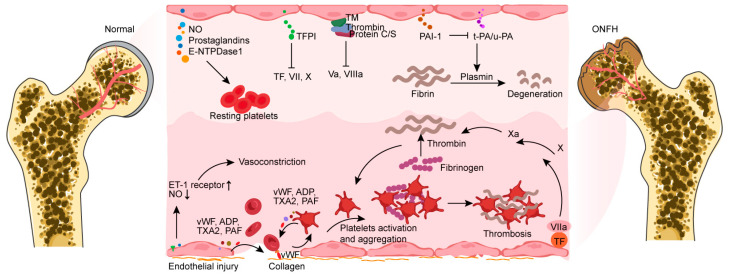
Endothelial cells share several molecules that target platelets or coagulation factors, forming an anticoagulant and antithrombotic barrier surface. Vasoconstriction occurs because of endothelial injury exposing subendothelial collagen and an imbalance of vasoactive chemicals. Collagen and platelets combine to form a connection complex with endothelial-derived vWF, which facilitates platelet activation. The factors (vWF, ADP, TXA2, and PAF) produced from endothelial cells exhibit the capacity to directly activate platelets and promote their aggregation. Activated platelets can then produce these compounds to aid in the coagulation process. Furthermore, endothelial injury exposes and binds tissue factor, which combines with factor VIIa to promote fibrin formation. NO, nitric oxide; E-NTPDase1, ectonucleoside triphosphate diphosphate hydrolase-1; TFPI, tissue factor plasminogen inhibitor; TF, tissue factor; TM, thrombomodulin; PAI-1, plasminogen activator inhibitor-1; t-PA, tissue plasminogen activator; u-PA, urokinase-type plasminogen activator; ET-1, endothelin 1; vWF, von Willebrand factor; ADP, adenosine diphosphate; TXA2, thromboxane A2; PAF, platelet-activating factor; ONFH, osteonecrosis of the femoral head.

Following vascular injury, endothelial cells are involved in all significant hemostatic processes and confine thrombosis to particular regions where it is necessary to restore vascular integrity. Subendothelial collagen is exposed to circulating platelets and forms junctional complexes. Endothelial- and platelet-derived von Willebrand factor (vWF) further strengthens this connection and promotes platelet activation and aggregation [56]. Patients with primary ONFH exhibit lupus anticoagulants, anticardiolipin antibodies, the factor V Leiden mutation, the prothrombin gene G20210A mutation, the methylenetetrahydrofolate reductase (MTHFR) C677T gene polymorphism, and P1A1/A2 polymorphism in glycoprotein IIIa, along with decreased concentrations of proteins C/S and anti-thrombin III in comparison to healthy individuals [45,57,58,59,60,61]. These aberrant alterations can lead to coagulation disorders in osteonecrosis of the femoral head following one flaw or a combination of multiple flaws. Endothelial dysfunction and injury resulting from many risk factors, including oxidative stress, endoplasmic reticulum stress, apoptosis, and necrosis, work in concert with coagulation abnormalities to exacerbate osteonecrosis in secondary ONFH [45,62]. By augmenting PAI-1 levels and diminishing tPA levels, GCs provoke an increase in coagulation and reduce fibrinolytic activity [63,64]. In patients and animal models of GIONFH, a great deal of fibrinogen and lipoprotein imply a hypercoagulable environment at the site of bone lesions [65,66]. A great deal of fibrinogen and lipoprotein results in activated platelets and retarded thrombolysis, further supporting the notion that thrombophilia and low fibrinolysis are closely associated with disease progression in osteonecrosis [67]. Thrombosis development increases intraosseous venous pressure and decreases arterial blood flow, resulting in hypoxia-induced ischemic damage in the bone vasculature. Immune activation and inflammatory responses simultaneously act on the endothelium to address the ensuing damage and orchestrate the healing process (Figure 1).

## 4. Endothelial Injury and Inflammation

### 4.1. Endothelial Injury Promotes Inflammatory Activation

The vascular endothelium actively participates in maintaining vascular homeostasis by balancing vasoconstrictor and vasodilator substances, anticoagulant and procoagulant substances, inflammatory and anti-inflammatory molecules, oxidants and antioxidants, and profibrinolytic and antifibrinolytic substances [68]. Endothelial dysfunction occurs as a reaction to different biochemical and physiological triggers, such as abnormal hemodynamic forces, excessive intramedullary compression, oxidative stress, intracellular injury, hyperlipidemia, harmful chemicals, and bacterial and viral infections [69,70,71]. Tiny crystals of cholesterol, monocytes, and lymphocytes reach the blood vessels and provoke an inflammation reaction that aids in the emergence of fatty streaks, leading to plaque deposition, progression, and collapse [72]. Plaque collapse promotes thrombosis, which, in conjunction with the blood clotting process, leads to atherosclerosis and vascular ischemia. Endothelial dysfunction is identified as both an evaluation and a predictive indicator for the advancement of atherosclerotic plaques throughout their stages of beginning and progressing, with the most adverse result being plaque collapse [73].

Multiple risks contribute to the production of reactive oxygen species (ROS) in the lining of vessels, such as glucocorticoids, drinking alcohol, hypercholesterolemia, diabetes, and hypertension [74,75,76]. Endothelium activity is impeded by ROS as they increase oxidative stress [77]. ROS accumulation in blood vessels leads to the production of harmful peroxynitrate and dysfunctional superoxide, disrupting the paracrine regulation of circulatory tone, antithrombosis, and vascular smooth muscle proliferation in endothelial cells [78]. Consequently, endothelial dysfunction brought on by oxidative stress propagates vasospasms, atherosclerosis, and vessel inflammation [79].

Inflammatory activation is crucial in initiating vasculopathy, which deteriorates due to both the synergistic effect on inflammation and endothelial injury [80]. Endothelial cells react to injury by releasing pro-inflammatory molecules, including interleukin 1β (IL-1β), interferons, tumor necrosis factor α (TNF-α), colony-stimulating factors, and vascular adhesion molecule 1 (VCAM-1) [81]. These molecules motivate monocytes and neutrophils to migrate to the activated endothelium surface and further promote inflammation, resulting in acute and chronic alternations in vascular permeability and vascular swelling [82]. Endothelial injury coincides with inflammation, revealing a hazardous process, including the mobilization of monocyte/macrophage cells through the circulatory system to the lesion, microcholesterol crystal translocation, lipid-containing cell blebbing, and cytokine/chemokine production [83]. These strongly promote the development of the plaque skeleton, leading to the rupture of structurally unstable plaques, the release of large amounts of thrombogenic substances into the lumen, and the triggering of atherosclerotic obstruction [84]. Even when the plaque is stable, endothelial apoptosis caused by plaque surface lesions can promote thrombosis [85].

### 4.2. Continuous Activation of Inflammation Hinders the Repair of Osteonecrosis

The initial goal of inflammation is to eliminate unwanted stimuli or infections and facilitate tissue healing. The inflammatory response facilitates the recruitment of factors that remove necrotic bone tissue and intramedullary tissue [86]. These proinflammatory factors derived from damaged blood vessels recruit macrophages, neutrophils, and other immune cells to remove harmful stimuli and relieve inflammation [87,88]. Normally, the resolution of inflammation facilitates tissue regeneration. However, in osteonecrosis, endothelial dysfunction coordinates inflammation and stimulates local immune cells to secrete inflammatory factors, inducing continued inflammation and hindering bone repair [89]. Vascular debris formed by a damaged endothelium and unstable plaques drives macrophages and neutrophils to secrete inflammatory factors, promoting the formation of immune thrombosis [90]. The injured endothelium, activated platelet-derived factors, and disrupted extracellular matrix serve as sources of damage-associated molecular patterns (DAMPs) [91]. Various immune cells, such as phagocytes, antigen-presenting cells, monocytes, and neutrophils, sense these stimuli through recognition receptors (PRRs) and further activate the inflammatory cascade and promote immune thrombosis, which in turn further destroys the injured endothelium [92].

#### 4.2.1. Neutrophils and NETs Induce a Procoagulant Phenotype in the Endothelium

**Figure 2:**
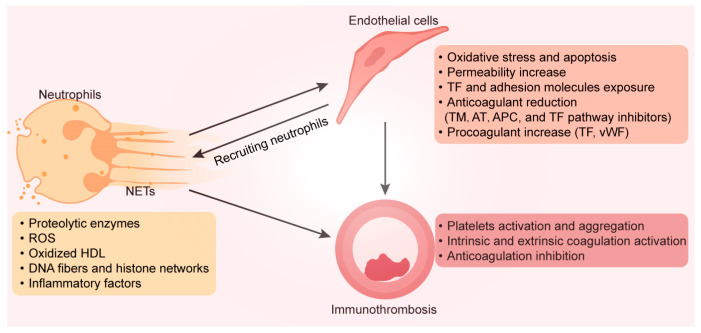
Neutrophils stimulated by bone lesion sites can produce NETs, consisting of a mesh-like structure made of DNA and chromatin, filled with various bioactive compounds (such as proteolytic enzymes, ROS, oxidized HDL, and inflammatory factors). These substances stimulate endothelial cells to exhibit a procoagulant phenotype. Adhesion molecules and tissue factors on endothelial cell surfaces recruit more immune cells, intensify inflammation, and enhance platelet activation and aggregation. This NET-mediated activation of the coagulation pathway and anticoagulation inhibition is called immunothrombosis. NETs, neutrophil extracellular traps; ROS, reactive oxygen species; HDL, high-density lipoprotein; DNA, deoxyribonucleic acid; TF, tissue factor; TM, thrombomodulin; AT, antithrombin; APC, activated protein C; vWF, von Willebrand factor.

As the most plentiful white blood cells in the blood, neutrophils play an essential role in innate immunity [93]. Neutrophils typically have the lowest lifespan among white blood cells, which is critical for avoiding continued inflammation and tissue healing in patients with osteonecrosis [94]. A considerable portion of neutrophils depart from the bone marrow during inflammation. Accompanying this process are the secretion of inflammatory cytokines and molecules, the synthesis of reactive oxygen species, and the release of proteolytic enzymes [95]. Moreover, proinflammatory substances and other enzymes found in neutrophil extracellular traps (NETs), which are released by active neutrophils, cause endothelial dysfunction and apoptosis [90]. The externalization of extracellular matrix proteases during NET formation causes endothelial injury and glycocalyx shedding, which exposes adhesion receptors and TF, thereby stimulating platelet and leukocyte adhesion and further inflammation [95]. The ROS-generating enzymes NO synthase (NOS) and oxidized high-density lipoprotein are also expressed at high levels in NETs, where they reduce their ability to efflux cholesterol and prevent foam cell formation, which fosters plaque formation and coagulopathy [96]. Engelmann et al. evaluated the mechanisms of NET-mediated thrombosis and used the term “immunothrombosis” to describe this process [97]. By producing a cascade of favorable feedback that enhances thrombosis both in vivo and ex vivo, the scaffold formed by DNA fibers and histone networks in NETs attracts platelets, leukocytes, red blood cells, and plasma proteins [98]. Inflammation in injured endothelial cells results in a reaction secretion (e.g., adhesion molecules and pro-inflammatory factors) during the acute phase, which disseminates to the circulation [99]. Through interactions with vWF, extracellular DNA in NETs can intensify thrombosis and inflammatory processes [100]. NETs stimulate the intrinsic coagulation process and facilitate the connection among neutrophils with factor XII (FXII). TF expression and activity in endothelial cells can be increased by NETs, expanding the extrinsic coagulation pathway [101]. Moreover, endothelial-derived anticoagulants such as antithrombin (AT), activated protein C (APC), and TF pathway inhibitors are inhibited by NETs, which leads to thrombosis [102]. In patients with ONFH, the femoral head microvessels contain a considerable number of neutrophils and NETs [103]. Osteonecrosis was further promoted by the intravenous injection of neutrophils capable of forming NETs in rats [104]. Therefore, the efficient removal of activated neutrophils is a potential target for the treatment of osteonecrosis (Figure 2).

#### 4.2.2. Endothelial Cell Junctions Coordinate Macrophage Activation

Macrophages play a crucial part in the inflammatory process by providing growth factors, cytokines, chemokines, and proteolytic enzymes. They also help combat infection, minimize tissue damage, and promote and support tissue remodeling [105]. Under healthy conditions, endothelial cells produce a range of anti-inflammatory mediators to prevent immune cell attachment to endothelial cells and vascular leakage [106]. Various adhesion molecules are released by endothelial cells in response to diverse stimuli in order to recruit monocytes to migrate towards the site of the lesion. This robust adherence results in polarization, which refers to alterations in morphology and phenotype [107]. Liu et al. reported that endothelial-derived extracellular vesicles promote the M1 polarization of macrophages and persistent inflammation by activating the TLR4/NF-κB signaling pathway, which may be a prerequisite for macrophage-induced chronic inflammation in GIONFH [108]. Endothelial cell adherent junctions are compromised, and endothelial cell connections become unstable when macrophages residing in bone lesions are stimulated by persistent inflammation associated with osteonecrosis to secrete inflammatory substances that disrupt these junctions [109,110]. The compromise of endothelial barrier function caused by this instability gives rise to the unregulated migration of monocytes and macrophages, as well as vascular leakage. Along with vascular abnormalities, endothelial cell conjunction defects can also cause vessel fragility and rupture, as well as the emergence of bleeding and edema [111]. Angioedema, cancer, diabetic microangiopathy, and allergic reactions are vascular conditions where a common characteristic is a breakdown of the barrier [112]. Endothelial connections facilitate attachment and initiate intracellular signals that determine cell location, limit development, and promote apoptosis [111]. They are crucial for maintaining vascular integrity and homeostasis.

#### 4.2.3. Adaptive Immune Cells

Adaptive immune dysfunction plays an important role in osteonecrosis, but its effect on blood vessels has rarely been investigated. Lymphocytes, particularly T cells, have an essential role in atherosclerosis, suggesting that they may be involved in vascular damage in osteonecrosis [113]. Different T-cell types in the area of bone lesions modulate the inflammatory environment and endothelial homeostasis. For instance, mice with osteonecrosis showed a large decrease in the amount of circulating Treg cells, which can dramatically lower plaque inflammation; however, T-helper (Th) cells and cytotoxic T lymphocytes (CTLs) accelerated the development of osteonecrosis [114,115,116]. Regulatory T cells (Tregs) release many anti-inflammatory substances, including IL-4, IL-10, and TGF-β, which can relieve inflammation in bone lesion areas [117]. Osteonecrosis of the jaw and ONFH are associated with a large increase in circulating Th17 cells, which produce IL-17 to sustain persistent inflammation [115]. IL-17 may cause atheroma development and blood vessel injury by recruiting neutrophils. CTLs can cause endothelial toxicity or damage through the production of cytokines.

Abnormal B-cell function and quantitative changes are also associated with vascular deterioration in patients with osteonecrosis [116]. B cells play both protective and pathogenic roles in plaque formation [118]. Low-density lipoprotein (LDL)-neutralizing and oxidized LDL-neutralizing antibodies (mainly natural IgM antibodies) protect against atherosclerosis, while other antibodies (mostly IgGs) enhance the inflammatory response to LDLs via macrophages [119]. Anticardiolipin antibodies are present in 30–40% of nontrauma ONFH patients, which partially explains the abnormal B-cell function associated with vascular thrombosis and osteonecrosis through damage to endothelial cells [120,121]. Autoantibodies promote the formation of neutrophil NETs [95]. B cells exhibit improved antigen presentation, and Th cell-secreted cytokines, including interferon-γ, further induce B cells to generate autoantibodies and induce endothelium injury in ONFH [122]. Regulatory B cells (Bregs) restrict immune cells from expanding, especially T lymphocytes, by producing IL-10, IL-35, and TGF-β [123]. More development in favorable B-cell types may be advantageous for osteonecrosis treatment, yet this is a challenge that has to be explored further.

## 5. Conclusion and Future Perspectives: Targeting the Endothelium to Treat Osteonecrosis

Currently, the vast majority of patients with ONFH ultimately require total hip arthroplasty surgery. Joint preservation is still a better option than joint replacement for younger patients, even if total hip arthroplasty can greatly enhance a patient’s quality of life due to numerous problems, including hip dislocation, periprosthetic fractures, and prosthetic loosening [124]. There are many treatments for early ONFH, such as core decompression, extracorporeal shock wave therapy, vascularized bone transplantation, and stem cell therapy combined with vascularized bone transplantation [125,126,127]. These treatment strategies can significantly reduce the volume of bone lesions, delay the progression of osteonecrosis, and relieve pain, but they cannot completely cure osteonecrosis. Therefore, it is crucial to improve microcirculation disorders of the femoral head as early as possible and restore the blood supply. This study summarizes recent endothelial-targeted treatments for osteonecrosis, including alleviating endothelial dysfunction, improving coagulopathy, alleviating inflammation, and promoting angiogenesis (Table 2).

Type-H vascular endothelial cells couple the osteogenic and angiogenic processes in bone development, regeneration, and repair. The description of type-H vessels provides a deeper comprehension of the molecular and biological mechanisms behind the communication between endothelial cells and osteolineage cells. Bone loss diseases such as osteoporosis, fractures, and osteonecrosis are closely related to type-H vascular variations. Numerous treatment approaches aimed at type-H vessels have demonstrated noteworthy therapeutic outcomes in certain animal models of bone disease. Injecting recombinant PDGF-BB, for instance, targets type-H angiogenesis, hence promoting fracture healing and relieving osteoporosis. The pathophysiology of these disorders may vary in humans and animal models, as these findings have mostly focused on mouse disease models to date. More human ONFH specimens and animal models are required to elucidate the distribution of H-subtype blood vessels in the necrotic, sclerotic, and normal portions of ONFH because of the insidious character of early ONFH.

Early and consistent anticoagulant therapy for primary ONFH can delay osteonecrosis from progressing, lessen or eliminate discomfort, and enable the patient to resume full activity and range of motion. Nearly all patients experienced symptom relief, full function was restored, and femoral head collapse was avoided. Anticoagulation therapy can protect joints from progressive osteonecrosis, thereby reducing the need for total hip replacement. Although a prospective study noted that anticoagulation therapy may have little effect on patients with secondary osteonecrosis, anticoagulation therapy can still significantly alleviate GIONFH in some animal models.

Necrotic bone undermines local immune function, causes unchecked inflammation, produces a chronic inflammatory milieu, and impedes bone regeneration and repair along the course of osteonecrosis. At present, the important regulatory role of immune cells is mainly reflected in the abnormal repair process initiated by the femoral head after osteonecrosis occurs. It cannot be ruled out that immunological abnormalities and chronic inflammation mediated by endothelial dysfunction exist prior to the development of bone lesions, as endothelial dysfunction plays a significant role in the early stages of this disease. The structural deterioration or even collapse of the femoral head can result from abnormal healing brought on by chronic inflammation, which interferes with bone regeneration and repair. Thus, creating medications that are unique to the vasculature and targeting the endothelium are crucial for prospective osteonecrosis treatments.

## Figures and Tables

**Table 1:** Vascular endothelial cell-derived factors orchestrate bone homeostasis.

Factors	Targets	Effects	Mechanism	References
Exosome	MSC	Promotes osteogenic differentiation and inhibits adipogenic differentiation	Inhibits STAT1 Activates MAPK/ERK pathway	Li et al. [24] Wu et al. [25]
Lactate	MSC	Promotes osteogenic differentiation	Histone H3K18la lactylation	Wu et al. [30]
BMP	MSC and chondrocyte	Promotes osteogenic differentiation and bone mineralized matrix formation	Via SMAD pathway	Salazar et al. [26]
Sema3a	Osteoblast and osteoclast	Promotes osteogenesis and inhibits bone resorption	Regulates Rho A and Wnt/β-catenin pathway	Kim et al. [27]
PGE2	Osteoblast, MSC, and chondrocyte	Promotes osteogenic differentiation and cartilage degeneration	Activates cAMP pathway	Li et al. [28] Sun et al. [29]
Noggin	Osteoprogenitor and chondrocyte	Promotes bone formation and induces hypertrophic chondrocytes	Promotes SOX9 expression	Cheng et al. [32] Ramasamy et al. [33]
MMP9	Chondrocyte	Removes the cartilage matrix and resorbs the cartilage template	Coordinates with non-resorbing osteoclasts to promote bone elongation	Romeo et al. [34]

MSC, mesenchymal stem cell; STAT1, signal transducer and activator of transcription 1; MAPK, mitogen-activated protein kinase; ERK, extracellular signal-regulated kinase; BMP, bone morphogenetic protein; SMAD, small mothers against decapentaplegic; Sema3a, semaphorin 3a; PGE2, prostaglandin E2; SOX9, SRY-box transcription factor 9; cAMP, cyclic adenosine monophosphate; MMP9, matrix metalloproteinase 9.

**Table 2:** Targeting endothelial cells to treat osteonecrosis.

Therapy	Effects	Mechanism	References
Biochanin A	Attenuates endothelial dysfunction via an increase in ZO-1 expression and a decrease in ICAM-1 expression	Binds to its target proteins, AKT1 and TNF-α	Liu et al. [128]
Morroniside or Vitamin B2	Promotes tube formation, migration, and angiogenic protein expression in a rat model of GIONFH	Activates the PI3K/AKT pathway	Jiang et al. [129] Guo et al. [130]
MicroRNA-112-5p	Attenuates endothelial viability, migration, and tube formation and decreases the level of proinflammatory cytokines in GIONFH patients	Binds to GREM2 and inhibits its downstream BMP/SMAD signaling	Huang et al. [131]
MicroRNA-137-3p	Promotes angiogenesis and increases the number of circulating EPCs in a rat model of GIONFH	Targets RUNX2 and CXCL12	Kong et al. [132]
Extracorporeal shockwave	Ameliorates endothelial apoptosis and promotes the angiogenesis and proliferation in a rat model of GIONFH	Via the microRNA-135b/FOXO1 pathway	Wu et al. [125]
Dimethylox-alylglycine	Attenuates endothelial dysfunction in a rabbit model of GIONFH	Activates HIF-1α signaling	Shao et al. [133]
Luteolin or DNA aptamer	Ameliorates endothelial necroptosis and promotes angiogenesis and migration in GIONFH patients	Regulates the RIPK1/RIPK3/MLKL pathway	Xu et al. [134] Fan et al. [135]
Ginkgo biloba L. extract	Ameliorates endothelial apoptosis and promotes angiogenesis and migration in a mouse model of GIONFH	Activates the PI3K/AKT/eNOS pathway	Cao et al. [136]
Desferoxamine	Promotes angiogenesis in a rat model of GIONFH	Via the HIF-1α/VEGF pathway	Jing et al. [137]
VO-OHpic	Attenuates apoptosis and promotes the angiogenesis of EPCs in a rat model of GIONFH	Activates the Nrf2 pathway and inhibits the mitochondrial apoptosis pathway	Yao et al. [138]
Icariin	Promotes migration, proliferation, tube formation, and angiogenesis-related cytokine expression in a rat model of GIONFH	Activates AKT and BCL-2 Inhibits BAX Increases MicroRNA-335	Yu et al. [139] Yue et al. [140]
Pravastatin	Promotes the autophagy of EPCs and protects against GC-induced apoptosis	AMPK-mTOR pathway via LKB1	Liao et al. [9]
Enoxaparin	Improves hypofibrinolysis in patients with osteonecrosis	Decreases the level of PAI-1	Heydock et al. [141]
Modified Qing’e Pill	Promotes fibrinolysis in patients with ONFH	Induces higher adiponectin levels and lower vWF and PAI-1 levels	Li et al. [142]
Resveratrol	Reduces thrombosis in a rabbit model of GIONFH	Increases the level of thrombomodulin	Zhai et al. [143]
Anticoagulation treatment	Prevents progression in patients with early ONFH	Daily low-dose apixaban treatment controls the disease	Glueck et al. [8]
Vitamin C/E	Reduces apoptosis, endothelial dysfunction, and inflammation	Increases the level of HDL and decreases the level of GSH, TG, and TC	Beytemur et al. [144]

ZO-1, zonula occludens-1; ICAM-1, intercellular adhesion molecule-1; TNF-α, tumor necrosis factor-α; PI3K, phosphoinositide 3-kinase; AKT, protein kinase B; GIONFH, glucocorticoid-induced osteonecrosis of the femoral head; GREM2, gremlin-2; BMP, bone morphogenetic protein; SMAD, small mothers against decapentaplegic; EPCs, endothelial progenitor cells; RUNX2, runt-related transcription factor 2; CXCL12, C-X-C motif chemokine ligand 12; FOXO1, forkhead box protein O1; HIF-1α, hypoxia inducible factor-1α; RIPK, receptor-interacting protein kinase; MLKL, mixed-lineage kinase domain-like; eNOS, endothelial nitric oxide synthase; VEGF, vascular endothelial growth factor; Nrf2, nuclear factor erythroid-2-related factor 2; BCL-2, B-cell lymphoma-2; BAX, BCL2-associated X; VO-OHpic, 3-hydroxypicolinate vanadium; GC, glucocorticoid; AMPK, adenosine 5‘-monophosphate-activated protein kinase; mTOR, mammalian target of rapamycin; LKB1, liver kinase B1; PAI-1, plasminogen activator inhibitor-1; vWF, von Willebrand factor; HDL, high-density lipoprotein; GSH, glutathione; TG, triglyceride; TC, total cholesterol.
